# Introduction to the Special Issue: Theory and Practice of Connectivity in the Indo-Pacific—Spheres, Logics, and Regional Dynamics

**DOI:** 10.1007/s12140-023-09411-x

**Published:** 2023-05-16

**Authors:** Bart Gaens, Ville Sinkkonen, Anu Ruokamo

**Affiliations:** Finnish Institute of International Affairs (FIIA), Helsinki, Finland

**Keywords:** Connectivity, International order, Regionalism, Indo-Pacific

## Abstract

This short introduction to the Special Issue entitled “Theory and Practice of Connectivity in the Indo-Pacific – Spheres, Logics, and Regional Dynamics” serves four objectives. It first briefly outlines the rationale for theoretical and empirical engagement with the concept of connectivity, which has become a ubiquitous term in the policy parlance of key global actors in recent years. The introduction then provides a short leader on the connectivity initiatives of key players, specifically China, the USA, Japan, the European Union, and Russia, with a particular focus on the Indo-Pacific space. Third, the seven articles that comprise the Special Issue are summarised. The contributions include a theoretically and conceptually oriented lead article, which introduces an analytical framework for the study of connectivity, and six more empirically motivated contributions that draw upon the said framework. Finally, key takeaways arising from the articles with respect to a broader research agenda on connectivity are discussed.

## Introduction

*Connectivity*, the overarching theme of this special issue comprising seven articles, is obviously not a new phenomenon. Literally meaning “the quality, state, or capability of being connective or connected” [1], the word has been in existence since the 1890s. In the second half of the twentieth century, the concept gained prominence in different scientific fields, including in the world of telecommunications and computing, as, for example, in internet connectivity. However, it was only in the 2010s that connectivity became a buzzword in diplomacy, economic integration, and international relations. The concept is closely related to globalisation, but arguably without the negative connotation associated with that term. Connectivity is certainly also key to regional integration. The Association of Southeast Asian Nations (ASEAN), for example, is an organisation that focuses strongly on connections and infrastructure development as a tool for integration. The adoption of the organisation’s *Master Plan on ASEAN Connectivity* in 2010 was instrumental in bringing the term connectivity to prominence in discussions about international and global phenomena. ASEAN considers physical (e.g., transport, ICT, and energy), institutional (e.g., trade, investment, and services liberalisation), and people-to-people linkages (e.g., education, culture, and tourism) as key means to achieve an integrated ASEAN community [2: 8].

Today’s world is indeed more deeply interconnected than ever at all levels of society. Connectivity has developed with increasing speed in domains such as infrastructure development and transport connections; financial cooperation and customs, trade, and investment facilitation; information technology (IT) and digital links; energy networks; and people-to-people, educational, institutional, and social-cultural linkages. At the same time, in an age defined by ever more tense great power relations, a fracturing of the international order, and global crises that do not respect borders, we observe intensifying attempts to disrupt various connections. Countries can, for example, impede economic transactions through tariffs and sanctions with the explicit or implicit aim of disconnecting or stifle the movement of people and information across borders through various regulatory or coercive means. As a result of all of these developments, connectivity has become a ubiquitous and much-discussed term in policy circles as well as, increasingly, in the research community.

This short introduction to the Special Issue serves four purposes. It first briefly outlines the rationale for theoretical and empirical engagement with the concept of connectivity within the discipline of international relations (IR) and possibly more broadly. It then provides a short leader on the connectivity initiatives of the key global players, specifically China, the USA, Japan, the European Union, and Russia, with particular focus on the Indo-Pacific space. Third, we introduce and summarise the seven articles that comprise the Special Issue. These include a theoretically and conceptually oriented lead article, which introduces an analytical framework for the study of connectivity, and six more empirically motivated contributions that draw upon the said framework. Finally, we discuss key takeaways arising from the articles with respect to a broader research agenda on connectivity.

## The Rise of Connectivity: Theoretical and Practical Considerations

While connectivity has undeniably become a buzzword in international politics and is arguably turning into a key paradigm of the global system today, the notion is rarely defined with sufficient precision. To our knowledge, the most useful definition still comes from the world of policy, more specifically the Asia-Europe Meeting (ASEM)—a multilateral forum encompassing 51 states from Asia and Europe, along with the European Union and the ASEAN Secretariat. The November 2017 Foreign Ministers’ Meeting in Nay Pyi Taw, Myanmar, stipulated the following:

Connectivity is about bringing countries, people and societies closer together. It facilitates access and is a means to foster deeper economic and people-to-people ties. It encompasses the hard and soft aspects, including the physical and institutional social-cultural linkages that are the fundamental supportive means to enhance the economic, political-security, and socio-cultural ties [...] which also contribute to the narrowing of the varying levels of development and capacities [3].

This broad definition has the benefit of drawing attention to connectivity as a phenomenon that transcends the physical world and can develop across different levels of social organisation, from the inter- and transnational all the way to seemingly mundane people-to-people contacts.

In terms of academic debates, especially in the field of IR, zooming in on connectivity is important for at least four reasons. First and foremost, despite its ubiquity, the concept is under-theorised. By implication, the different manifestations of connectivity within the global arena, and their attendant implications for international cooperation and competition, are not sufficiently well understood. The conceptual vocabulary for capturing such phenomena also remains fragmented.

Second, a focus on connectivity arguably makes it possible to move beyond often uninformative debates on the balance of power capabilities and system polarities [4, 5]. Emphasising connectivity reveals how power is exercised with, as well as against, other actors through the continuous establishment, management, recalibration, and severing of connective relationships [cf. 6, 7, 8]. These mechanisms then create dynamic, transformative frameworks of action beyond any traditional capabilities- or polarity-based divisions of power.

Third, and relatedly, a focus on connectivity allows for understanding international order not as a descriptor for stability, but as a purposively constructed entity that actors on different levels of social organisation can shape via *intentional* agency [9, 10]. Actors, whether states, international organisations, cities, corporations, or in some cases even individuals can, and are inclined to, create a multitude of connections for *functional* ends. This is related to newer understandings of the international arena as “multi-ordered” [11, 12] or defined by “multiplexity” [13]. Through connections, actors can opt into or out of different components of international and regional order(s), creating opportunities for both increased convergence and divergence.

Fourth, connectivity has implications for how regions are constructed and how they should be conceptualised. Following trade-centred regionalisation, politically oriented neoliberal regionalism, and a phase marked by proactive transregional cooperation [e.g., 14, 15], connectivity arguably heralds the advent of a novel, “fourth generation” of regionalism. Under this notion, regions are increasingly defined by spatial fuzziness and the construction of functional links that actors manage pragmatically in order to pursue both interest- and identity-based ends. The new concept of the Indo-Pacific, transcending previous socially constructed regional boundaries, is a significant case in point [16].

At a more empirical and practical level, it is likewise evident that an urgent need exists for more thorough studies on connectivity in all its dimensions. One can surmise at least four important motivations. First, connectivity plays a vital role in addressing the continuing global need for infrastructure investments, in boosting trade and investment, and in bringing the peoples of the world closer together. According to the World Economic Forum, almost 14% of global GDP is invested in infrastructure; however, as the global population expands, urbanisation and economic development proceed, and existing infrastructure gradually crumbles, much more investment is needed, to the effect of $15 trillion by 2040 [17]. This is essential to provide electricity for unserved populations, while mitigating climate change, or to make information and communications technology (ICT) services widely available to all layers of society.

Second, connectivity and sustainable development are strongly interlinked. Connectivity initiatives and projects need to contribute to the realisation of the 2030 Agenda for Sustainable Development, including the 17 Sustainable Development Goals (SDGs), adopted by all United Nations Member States. Connectivity in all its forms has to be rooted in economic, fiscal, environmental, and social sustainability, in line with international standards and based on key principles such as a level playing field, free and open trade, inclusiveness, fairness, and transparency.

Third, we are currently witnessing a connectivity race. In Asia, China has adopted a leading role in driving forward connectivity. China’s Belt-and-Road Initiative, adopted in 2013 and launched in 2014, has become the overarching brand for Beijing’s attempts to invest in large-scale infrastructure projects from Asia to Europe, including through land bridges and maritime transport corridors. It includes the Silk Road Economic Belt, stretching from China to Europe, and the Maritime Silk Road promoting shipping routes from China to Europe, through Southeast Asia, India, and Africa. This has prompted other countries and regional organisations to devise their own connectivity strategies, ideally yielding opportunities for cooperation and leading to partnerships and “infrastructure alliances”.

Fourth, a strong element of competition often underlies connectivity, as key actors aim to establish contending spheres of interest, for example through infrastructure development. For some, the interconnected infrastructure of the global economy is increasingly replacing conventional warfare as the battleground of conflict [see, e.g. 18]. At times, we can even speak of “connectivity wars” that play out through (geo)economic warfare, economic statecraft, the weaponization of international institutions, and infrastructure competition [19]. It is no exaggeration to state that connectivity is increasingly becoming an area of great-power competition involving states like China, the USA, Japan, India, Russia, and also the EU.

As connectivity has become the key notion informing current policy strategies of the world’s major actors, the prime aim of this Special Issue is to make sense of the objectives and wider ramifications of these connectivity pursuits, particularly in terms of region-building efforts in the Indo-Pacific and Europe. In addition to helping us understand how regional (dis)orders currently come into being and interact, the contributions in this Special Issue study the conceptual underpinnings of connectivity and relate the notion to other central mechanisms of power politics. This research agenda is urgently important given the precarities of the current global constellation, permeated by a range of different crises, from the war in Ukraine to various climate tragedies. These risk eroding the transregional and international frameworks of connective cooperation established during the post-Cold War era.

## Key Connectivity Players: Proliferating Initiatives in an Age of Flux

To set the stage, it is useful to take a brief look at the connectivity initiatives of the key connectivity players covered on the pages of this Special Issue. Such an exposition needs to start with the Belt and Road Initiative (BRI), in view of China’s driving role in setting off what amounts to a global connectivity race. This grand Chinese connectivity initiative also factors, in one way or another, in all of the author contributions.

In 2013, Xi Jinping published plans for the One Belt, One Road (OBOR), which later came to be known as the Belt and Road Initiative (BRI) [20: 169]. From its early focus on traditional infrastructure projects, the BRI has extended to also include, for example, the Digital [21], Space [22], Polar [23], and Health Silk Roads [24].

In its premise the BRI connectivity project was set up to support Beijing’s economic needs stemming from domestic overcapacity [20: 173], as well as strategic objectives in connection to China’s “peripheral diplomacy” [25: 3]. The initiative also has ideational motivations as demonstrated by Kallio [26] in this special issue. The BRI is comprehensive but also amorphous, allowing for a wide variety of projects to be brought under the BRI rubric [25: 2, 27: 6]. At the time of writing, over 140 countries are in some way affiliated with the initiative [28]. According to estimates, China has already invested over 350 billion through the BRI since 2013. The value of construction projects has exceeded 530 billion [27: 6, 29].

After a great deal of interest towards the initiative during its first years [27: 3, 30], the support for BRI projects has started to dwindle, both domestically and internationally. Partner countries have become increasingly concerned over the so-called “debt trap diplomacy” and the sustainability of Chinese loans, as well as the overall environmental and socio-economic implications of the BRI projects [30, 31]. The economic impact of the COVID-19 pandemic has further highlighted the need for changes [30, 31], and there seems to be a new kind of emphasis from Beijing on the quality and sustainability of the projects [27: 6, 30].

In addition, the different modes of BRI connectivity have started to become more prominent. Amid the COVID-19 pandemic, Beijing highlighted the Health Silk Road aspect of the BRI, linking deliveries of protective equipment and, later on, Chinese vaccines to the initiative [32]. The focus on the Digital Silk Road is another example of readjustments that allow a move away from the traditional heavy infrastructure projects towards less resource-intensive undertakings in the digital domain [27: 7]. In 2021 and 2022, Xi Jinping also publicised three new initiatives, the Global Development Initiative (GDI), the Global Security Initiative (GSI), and, most recently, the Global Civilisation Initiative (GCI) [33: 1]. The new initiatives could diversify China’s development cooperation and bring new programs to the foreground [27: 7, 34]. They might also just be a way to repackage old foreign-policy goals under a new name, and, according to official Chinese accounts, the GDI has not been set up as a substitute for the BRI [35, 36]. The fact that the BRI remains Xi Jinping’s “signature initiative”, carved into the Party constitution [27: 4], is an indication that the Belt and Road Initiative is unlikely to be going anywhere anytime soon [25: 2]. This means other key actors will continue to frame their connectivity forays as explicit or implicit responses to the BRI into the foreseeable future.

The USA’s and Japan’s connectivity activities are linked to the “Free and Open Indo-Pacific” (FOIP) rubric [37: 2]. An idea initiated by Japan in 2016 and adopted by the USA in 2019 [38, 39: 6], FOIP is widely viewed as an effort to offset China in the Indo-Pacific (although Japan has refuted this [38]). In the summer of 2021, the USA together with the G7 put forth the Build Back Better World (B3W) initiative. B3W centres on four areas: climate, health and health security, digital technology, and gender equity and equality [40]. The initiative has been depicted as focusing on values, high standards, and transparency—an implicit jab at the BRI [37: 15]. The B3W was reintroduced as the Partnership for Global Infrastructure and Investment (PGII) in 2022, which has promised $600 billion to infrastructure development between 2022 and 2027 [41]. Compared to the B3W, and in more direct competition with the BRI, the PGII includes greater attention to more traditional infrastructure projects [42]. Despite being launched with fanfare, the B3W made only slow progress in the connectivity sphere; whether the PGII can change this course remains undetermined [37: 18–19].

As a member of the G7, Japan is also part of the PGII. Japan’s own connectivity projects were initially launched under the banner of the Partnership for Quality Infrastructure (PQI) of 2015 [43], and its core values formed the template for the adoption of the Principles for Quality Infrastructure Investment (PQII) by the G20 in 2019 [44]. Similar to the connectivity initiatives of other major players, the PQI has been viewed as Japan’s response to the BRI [45]. In its infrastructure development, Japan has traditionally focused on Southeast Asia. However, under the FOIP umbrella, the geographical reach of Japan’s connectivity forays has expanded to encompass also South Asia and the Pacific Islands as well as Africa [37: 21–22]. The PQI was originally set to invest $110 billion in connectivity projects. As the geographical reach of the partnership has expanded, the budget was raised to $200 billion [46].

The European Union published its connectivity initiative, the “Global Gateway”, in 2021, planning to invest €300 billion by 2027. The Global Gateway seeks to advance “smart, clean and secure links in digital, energy and transport sectors and to strengthen health, education and research systems across the world” [47]. The initiative is seen by many as the EU’s response to China’s Belt and Road Initiative, as it is said to follow principles such as “democratic values and high standards”, “good governance and transparency”, and “equal partnerships” [see 48]. The initiative is a continuation of the EU’s connectivity strategy from 2018. However, compared to the earlier strategy, the Global Gateway is more concrete in its funding plan. Also, whereas the 2018 strategy focused specifically on Asia and the Indo-Pacific, the Global Gateway has a much wider remit [48].

Russia’s connectivity efforts, particularly with respect to Asia, have encompassed three areas: the Russian Far East, Central Asia, and the so-called Greater Eurasia. Per Kaczmarski and Silvan [49] in this Special Issue, only the Greater Eurasian Partnership endeavours to counter connectivity efforts by the USA, for instance in the Indo-Pacific, as well as seeking to raise Russia’s status vis-à-vis China. Other connectivity efforts in the Russian Far East are linked to regional development and connectivity in Central Asia, “an attempt to slow down the erosion of Soviet connectivity legacy” [49: 3]. However, Russia does not have a consistent approach when it comes to connectivity. Its actions in the connectivity sphere “reflect primarily political rather than economic reasoning” [49: 3], causing the efforts to have less actual impact.

This brief overview of key players’ connectivity initiatives attests to the ubiquity of the phenomenon and underlines how states have embraced connectivity as a way to not only profit economically and further regional development and cooperation, but also as part of grand strategy formation in an age defined by increasingly tense great-power relations and cascading crises. To make further sense of these potentially incongruent drivers, we now turn to the contributions of the articles in this Special Issue.

## Exploring the Logics and Spheres of Connectivity: the Contributions to the Special Issue

The first article of the Special Issue by Bart Gaens, Ville Sinkkonen and Henri Vogt [50] lays out a novel analytical framework for the study of connectivity. The article starts from the premise that global and regional orders are increasingly defined by various types of connective links that are purposively constructed by intentional actors that reside at different levels of social organisation. There is thus an element of *functionality* to actors’ connectivity forays. To grasp the totality and nuance of connectivity as a phenomenon, the article introduces an analytical framework composed of six connectivity logics and six connectivity spheres.

In terms of the logics, *cooperation* entails the creation of inclusive connections based on absolute gains, *copying* the emulation of “best” connectivity practices or regulatory frameworks, and *cushioning* the reduction of risks by establishing connections with various connectivity actors. *Contestation*, in turn, refers to actors establishing connections to gain advantages over competitors, and *containment *means shutting out others through disconnection or establishment of exclusionary connectivity spheres. *Coercion*, finally, sees actors forcing others to connect in a particular way or refrain from connecting entirely. The authors also argue that these logics reside on a continuum when it comes to implications for international and regional order(ing), cooperation being the most constitutive of order and coercion being the most corrosive. The logics ultimately play out differently within the six connectivity spheres, which are termed *infrastructural*, *economic* and financial exchange, *institutional* frameworks of governance, *knowledge* exchange, *socio-cultural*, and *security*. The article illustrates the interplay between logics and spheres through a collection of empirical examples drawn from the Indo-Pacific region. The other articles of the Special Issue draw and reflect upon this toolkit, each in their own way.

The second article, authored by Jyrki Kallio [26], zooms in on the ideational motivations behind China’s BRI. In Kallio’s [26] view the BRI should not be viewed merely as a connectivity initiative aimed at enhancing Beijing’s geopolitical or geoeconomic influence, but as part of the ideological *contestation* over institutions and values that China is engaged in with the USA and the broader West. In this manner, BRI must be viewed in the context of broader visions such as the “Community of Common Destiny”, ordering alternatives like “Whole-Process People’s Democracy” and the ancient concept of *Tianxia*, literally meaning “all-under-Heaven”. In this manner, Kallio underlines the institutional and socio-cultural dimensions of China’s manifold connectivity projects that have been lumped under the BRI rubric, which appears more as an ambiguous (albeit ambitious) slogan rather than a strategy in any meaningful sense. Yet, looking beyond official rhetoric, it is hard to ignore that the BRI bears the potential of creating a “particularistic universe” around China in its neighbourhood—perhaps tantamount to ideational and ultimately physical containment of other great powers. Nevertheless, Kallio is ultimately sceptical of the “soft power” dividends or ideational pull of the BRI. The involvement of countries in connectivity projects, especially in China’s neighbourhood, appears to be driven by economic rationales as opposed to the attractiveness of Beijing’s “hollow” slogans. This may yet work in the “West’s” favour, as the competition over connectivity in the Indo-Pacific and further afield intensifies in the coming decades.

In order to gauge local reactions to China’s foreign investments, the third article in this Special Issue examines the process of “riskification” of Chinese Foreign Direct Investment (FDI) in four Nordic countries (Finland, Sweden, Denmark, and Norway). Mikael Mattlin and Mikko Rajavuori [51] argue that a causal narrative has emerged in these countries that stresses the potential strategic motivations and associated security risks of Chinese state investments. These perceptions have set in motion a process of legislative as well as policy-related changes. The authors conclude that FDI as an important dimension of cross-border connectivity is significantly affected by this “riskification” process. Investment monitoring is becoming the norm in Europe—arguably a means to contest or contain Chinese influence—even if its implementation is subject to the local economic and political context as well as prior legislation and policies. The authors contend that changed perceptions vis-à-vis Chinese investments are not only shaped by the greater attention given to the nature of the Chinese party-state and the potential strategic exploitation of investments by state-owned enterprises, but also by closer security ties to the USA and the UK, as well as by new EU FDI rules. Hence, connectivity in the security sphere has implications for economic and financial exchange as well as for the regulatory frameworks undergirding connectivity.

In the fourth article, Bart Gaens and Ville Sinkkonen [37] explore how the USA and Japan have sought to formulate a response to the BRI under the banner of the Free and Open Indo-Pacific (FOIP). In an effort to contain growing Chinese influence in the Indo-Pacific region, the USA has rolled out a large number of connectivity initiatives. However, these have been impacted by domestic politics, slow implementation, indecisiveness on whether to compete directly with China’s BRI in infrastructure development, and values-based conditionalities. Japan, for its part, has aimed to promote “quality infrastructure”. Tokyo’s strategy has been strongly rooted in securitized development cooperation and a focus on economic infrastructure development buttressed by technical support. Yet cooperative efforts with other actors have been slow to take off. Contestation with China drives Japan’s connectivity endeavours, and Japan’s stance has been pragmatic in spite of lip-service paid to values and norms. Based on the assessment of Japanese and US connectivity strategies, Gaens and Sinkkonen make a threefold argument: first, there exists a clear need to bring rhetoric and capabilities in line; second, Western actors need to crystallise their ultimate objective (compete with and potentially contain China or focus pragmatically on complementarities and comparative advantages); and third, the USA and Japan need to prioritise projects and spheres that are strategically important.

The fifth article by Tyyne Karjalainen [48] focuses on the EU’s connectivity policy, in particular the Global Gateway, analysing it in the context of the Union’s norm diffusion. While Karjalainen points out that the Global Gateway is novel in its attempt to put the Union’s “money where its mouth is” in order to compete with China’s BRI—or even perhaps contain it—she is interested in how the Union’s normative power is exercised through connectivity initiatives. Here, Karjalainen finds an actor talking the talk of partnership and cooperation, on the one hand, but unwilling to walk the walk, on the other. The EU is engaged in coercive connectivity to the extent that it is imposing its normative agenda, standards, and regulations upon partners that do not share or would otherwise be unwilling to accept the Union’s normative precepts. However, given the asymmetry in material capabilities, other states have little choice but to accept the Union’s normative offer (or, in terms of the analytical framework, partners lack cushioning options). In the process, Karjalainen not only bridges materialist and ideational understandings of connectivity, but also makes a theoretical contribution by bringing the much older debate on the EU’s normative power into the connectivity frame.

In the sixth article, Kristiina Silvan and Marcin Kaczmarski [49] analyse the strategy behind and implementation of Russia’s connectivity policy against the background of three distinct geographical spaces, namely the Russian Far East, Central Asia, and Greater Eurasia. The authors argue that Russia’s connectivity strategies have been fluid and need to be assessed at the subnational level. The logics of contestation, containment, and coercion are easily discernible vis-à-vis Europe and Russia’s post-soviet neighbours, while Russia’s approach to China has been marked by both containment as well as by cooperation and cushioning. In general, a wide gap exists between cooperative rhetoric and practical implementation. The copying logic can easily be witnessed in the Eurasian Economic Union modelled on EU legislation, and the Greater Eurasian Union based on the BRI mould. Russia’s war in Ukraine has put a brake on most connectivity efforts. However, China and Central Asian states’ willingness to extend connectivity projects with Russia indicates that the latter will remain a player in the field.

Finally, the seventh article by Katja Creutz [52] zooms in on Multilateral Development Banks (MDBs) as agents of connectivity. Focusing on the Asian Development Bank (ADB) and the Asian Infrastructure Investment Bank (AIIB), Creutz discusses the strategic, developmental, and financial agency of these banks. She argues the banks are not only connectivity “nodes” but also “agents”. On the one hand, they make it possible for other actors to connect and are, at the same time, linked to states’ (or their shareholders’) strategic visions. MDBs may thus feasibly be used as vehicles of cooperation with other countries, as well as means of contestation or even (implicit) containment, as has been the case with Japan’s upped contributions to the ADB after China introduced the BRI. At the same time, however, MDBs function as actors in their own right. They are development actors pushing for certain definitions of development with respect to, for instance, infrastructure, and also banks, concerned with questions like project viability, credit-worthiness, and attracting borrowers. By problematising connectivity agency, Creutz [52] comes to underline how connectivity straddles the private and public spheres, bringing attention to the different levels of social organisation on which connections and connective agency may unfold.

Taken together, the articles in this Special Issue demonstrate that connectivity strategies and policies in various spheres provide a plethora of empirical examples of the six logics identified by Gaens, Sinkkonen and Vogt [50], driving forward connective and disconnective efforts by the main global actors. The hard and soft infrastructures of connectivity provide numerous opportunities for cooperation between major players. However, a gap between strategies/rhetoric and capabilities/implementation often stands in the way of successful cooperative efforts. Emulation (copying) and hedging (cushioning), in particular by smaller states, are (most often) benign logics informing policies in the field of connectivity. However, on the darker side of the spectrum, the articles in this Special Issue also clearly show that connectivity has become a major site of (great-power) contestation, often incorporating logics of containment and even coercion. If rhetoric and capabilities are brought in line, resources are allocated to strategically important fields, and major actors focus pragmatically on complementarities and comparative advantages, connectivity can be a force for good, addressing the gap in infrastructure needs and promoting sustainable development standards. However, if competition continues to underscore connectivity strategies and their implementation, the Indo-Pacific region faces a fracturing into different, overlapping, and competing connectivity orders.

What remains obvious, however, is that connectivity initiatives on different levels will continue to proliferate in the coming decades. Just recently, in March 2023, Japan unveiled a new plan for a Free and Open Indo-Pacific, pledging more than $75 billion in public and private funds for new infrastructure investments across the region by 2030 and boosting the importance of “multilayered connectivity” as a key pillar and core element of the cooperation for FOIP. The Blue Dot Network—originally launched by the USA during the Trump era—is moving forward under the auspices of the OECD to provide a go-to certification mechanism for quality infrastructure investment. Given such swift evolution of the connectivity field, it is necessary for theoretical discussions and analytical tools to keep pace with the real-world developments. It is our hope that this Special Issue will serve as one significant signpost in facilitating deeper future engagement with connectivity, both conceptually and empirically. Such research undertakings do not merely accumulate vital knowledge on evolving connections. Given the ubiquity of connectivity, the phenomenon has potentially profound impacts on the daily lives of people around the world. A normative imperative therefore exists to pose difficult questions regarding the viability, sustainability, and adequacy of different connectivity initiatives with respect to local, regional, and global needs. This also means remaining constantly privy to the great- and regional-power interests underpinning different connectivity forays.
